# Combining pathological risk factors and T, N staging to optimize the assessment for risk stratification and prognostication in low-risk stage III colon cancer

**DOI:** 10.1186/s12957-023-03299-w

**Published:** 2024-01-04

**Authors:** Zhen-Yu Xian, Yi-Wen Song, Zong-Jin Zhang, Ying-Guo Gan, Yong-Le Chen, Tuo Hu, Xiao-Feng Wen, Tai-Wei Mo, Xiao-Wen He

**Affiliations:** 1 Department of Colorectal Surgery, Department of General Surgery, The Sixth Affiliated Hospital, Sun Yat-Sen University, Guangzhou, China; 2 Guangdong Provincial Key Laboratory of Colorectal and Pelvic Floor Diseases, The Sixth Affiliated Hospital, Sun Yat-Sen University, Guangzhou, China; 3 Biomedical Innovation Center, The Sixth Affiliated Hospital, Sun Yat-Sen University, Guangzhou, China; 4 Department of Radiotherapy, The Sixth Affiliated Hospital, Sun Yat-Sen University, Guangzhou, Guangdong China; 5 Department of General Surgery, The First Affiliated Hospital of Jinan University, Jinan University, 613 West Huangpu Avenue, Guangzhou, 510630 Tianhe District China; 6 Department of General Surgery, Guangzhou First People’s Hospital, No. 1 Panfu Road, Guangzhou, 510180 Yuexiu District China

**Keywords:** Colon cancer, Stage III, Pathological risk factors, Adjuvant chemotherapy, Prognosis

## Abstract

**Background:**

This study aimed to investigate the combined pathological risk factors (PRFs) to stratify low-risk (pT1-3N1) stage III colon cancer (CC), providing a basis for individualized treatment in the future.

**Patients and methods:**

PRFs for low-risk stage III CC were identified using COX model. Low-risk stage III CC was risk-grouped combining with PRFs, and survival analysis were performed using Kaplan–Meier. The Surveillance, Epidemiology, and End Results (SEER) databases was used for external validation.

**Results:**

Nine hundred sixty-two stage III CC patients were included with 634 (65.9%) as low risk and 328 (34.1%) as high risk. Poor differentiation (OS: *P* = 0.048; DFS: *P* = 0.011), perineural invasion (OS: *P* = 0.003; DFS: *P* < 0.001) and tumor deposits (OS: *P* = 0.012; DFS: *P* = 0.003) were identified as PRFs. The prognosis of low-risk CC combined with 2 PRFs (OS: HR = 3.871, 95%CI, 2.004–7.479, *P* < 0.001; DFS: HR = 3.479, 95%CI, 2.158–5.610, *P* < 0.001) or 3 PRFs (OS: HR = 5.915, 95%CI, 1.953–17.420, *P* = 0.002; DFS: HR = 5.915, 95%CI, 2.623–13.335, *P* < 0.001) was similar to that of high-risk CC (OS: HR = 3.927, 95%CI, 2.317–6.656, *P* < 0.001; DFS: HR = 4.132, 95%CI, 2.858–5.974, *P* < 0.001). In the SEER database, 18,547 CC patients were enrolled with 10,023 (54.0%) as low risk and 8524 (46.0%) as high risk. Low-risk CC combined with 2 PRFs (OS: HR = 1.857, 95%CI, 1.613–2.139, *P* < 0.001) was similar to that of high-risk CC without PRFs (HR = 1.876, 95%CI, 1.731–2.033, *P* < 0.001).

**Conclusion:**

Combined PRFs improved the risk stratification of low-risk stage III CC, which could reduce the incidence of undertreatment and guide adjuvant chemotherapy.

**Supplementary Information:**

The online version contains supplementary material available at 10.1186/s12957-023-03299-w.

## Introduction

Colon cancer (CC) is one of the most common human malignancies worldwide [1, 2]. There was significant heterogeneity in prognosis of stage III CC patients, with 30–40% of patients develop recurrence or metachronous metastasis after radical surgery [3]. Treatment options for stage III CC patients are still being refined to further improve the long-term prognosis of these patients.

The International Duration Evaluation of Adjuvant Chemotherapy (IDEA) study proposed that stage III CC could be divided into low-risk group (T1-3N1) and high-risk group (T4 and/or N2) based on T and N stages [4]. Based on data from the IDEA study, National Comprehensive Cancer Network (NCCN) guidelines have recommended that stage III CC patients should be stratified according to T/N stage to choose the regimen and duration of adjuvant chemotherapy [5]. For patients with low-risk stage III CC, 3 months of CAPEOX therapy or 3–6 months of FOLFOX therapy should be preferred [4, 5]. Pathological risk factors (PRFs) are the basis of stratification for prognosis and treatment guidance in stage III CC patients. In addition to T and N stages, PRFs including lymphovascular invasion (LVI), perineural invasion (PNI), and tumor deposit (TD) are associated with poor prognosis for stage III CC patients [6–9]. The prognosis of low-risk (T1-3N1) stage III CC patients with PRFs may not be “low-risk.” Risk stratification just based on T and N stages may lead to insufficient treatment for the selection of adjuvant chemotherapy duration in some stage III low-risk CC patients.

In this study, the prognosis of low-risk stage III CC patients was further evaluated combining with PRFs, and the risk stratification was optimized. The optimized risk stratification can provide reference for the selection of postoperative adjuvant therapy, which may reduce the incidence of insufficient adjuvant therapy in some low-risk stage III CC patients.

## Methods

### Patients and data collection

**Fig. 1:**
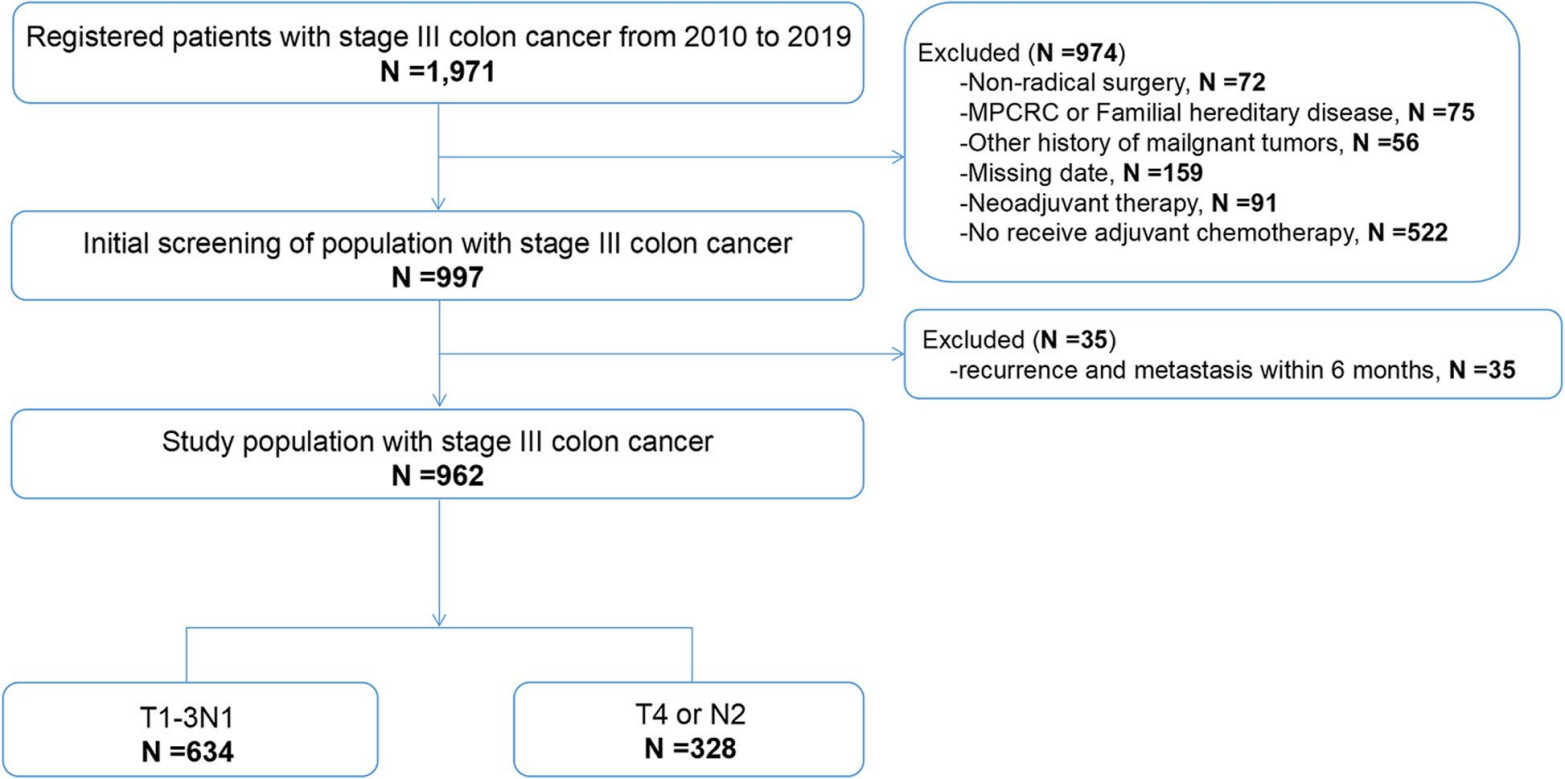
Flow chart of patient enrollment for this study (MPCRC, multiple primary colorectal cancer)

The data of stage III CC patients from 2010 to 2019 at the Sixth Affiliated Hospital of Sun Yat-sen University were retrospectively retrieved. Patients enrolled were diagnosed with colon adenocarcinoma, who received curative resection with postoperative pathological stage as III (Fig. 1). Exclusion criteria were as follows: (i) patients receiving surgery due to severe complications, (ii) multiple primary colorectal tumor or familial adenomatous polyposis, (iii) history of other neoplasms, (iv) patients received neoadjuvant therapy, (v) patients with missing data or lost visits, (vi) patients without adjuvant chemotherapy or adjuvant chemotherapy < 3 months, and (vii) follow-up period less than 6 months or metachronous recurrence and metastasis occurred within 6 months. Excluding patients with recurrence and metastasis within a 6-month time frame is justified due to research findings indicating that the definition of metachronous recurrence and metastasis should be established at least 6 months after the initial diagnosis of CC [10]. The study protocol was approved by the Ethics Review Committee of the Sixth Affiliated Hospital of Sun Yat-sen University (2022ZSLYEC-229).

In this study, a retrospective analysis was conducted based on data from the SEER databases (http://seer.cancer.gov/) to verify our hypothesis. Data of stage III CC patients from 2010 to 2015 were retrieved using SEER*Stat version 8.3.6. The selection criteria and screening process are illustrated in Supplementary Fig. 1.

### Clinicopathologic variables and objectives

Clinicopathologic variables extracted in this study were listed as follows: age, sex, pathological T stage, pathological N stage, tumor primary site, number of lymph nodes (LNs) harvested, tumor differentiation grade, tumor histological type, LVI, PNI, DNA mismatch repair (MMR) status, TD, and treatment method (including surgery method and adjuvant chemotherapy regiment). The outcomes included disease-free survival (DFS) and overall survival (OS). DFS was defined as the time from surgery until disease recurrence or death due to any cause, while OS was defined as the time from surgery to death of any cause. A second primary colorectal cancer was not considered as a DFS event.

### Statistical analysis

Continuous variables are presented as mean (standard deviation) or number (%), and categorical variables are presented as percentages. Statistical differences between two groups were analyzed using chi-squared tests for categorical variables. As for numeric variables, parametric tests were performed for numeric data subjecting to normal distribution, while nonparametric tests were utilized for numeric data not subjecting to normal distribution. The survival analysis was estimated by the Kaplan–Meier method and log-rank test. For factors associated with DFS and OS, Cox proportional hazard models were used to estimate hazard ratio (HR) and 95% confidence intervals (CI). A univariable Cox analysis was performed to assess the association between baseline characteristics and DFS/OS, and then, variables with *P* values < 0.1 were included in the multivariable Cox regression model.

For all statistical analysis, *P* value < 0.05 was considered as statistically significant. Data analysis and image plotting were performed using SPSS 26.0 software and GraphPad Prism 9 software*.*

## Results

### Patient characteristics

From 2010 to 2019, a total of 1971 stage III CC patients were screened in the Sixth Affiliated Hospital of Sun Yat-sen University. According to the exclusion criteria, a total of 974 patients were excluded, including 72 cases with non-radical surgery due to serious complications, 75 cases with MPCRC or familial adenomatous polyposis, 56 cases with other neoplasms, 159 cases with missing data or lost visits, 91 cases with neoadjuvant therapy, 522 cases did not receive postoperative adjuvant chemotherapy, and 35 cases with metachronous recurrence and metastasis within 6 months after operation. Then, 962 patients were found eligible for this study, of whom 634 (65.9%) were distributed in the low-risk group and 328 (34.1%) were divided into the high-risk group (Fig. 1).

There was no significant difference in age (*P* = 0.372) and sex (*P* = 0.629) distribution between stage III CC patients from a low-risk group and high-risk group. The incidence of mucinous or signet ring cell (23.8% vs. 14.8%, *P* = 0.001), LVI (34.8% vs. 14.5%, *P* < 0.001), PNI (33.2% vs. 20.3%, *P* < 0.001), and TD (45.1% vs. 36.6%, *P* = 0.012) were found to be more frequent in high-risk stage III CC patients comparing with those in the low-risk group. The proportion of patients in the high-risk group receiving adjuvant chemotherapy for more than 3 months was significantly higher than that in the low-risk group (58.8% vs. 41.2%, *P* < 0.001). The timing of receiving adjuvant chemotherapy after radical surgery in high-risk and low-risk groups was mostly within 6 weeks, and there was no significant difference (*P* = 0.184). The baseline characteristics of stage III CC patients between low-risk and high-risk groups were summarized in Table 1.

**Table 1:** Characteristics of stage III CC patients with low risk (T1-3 and N1) and high risk (T4 and/or N2) from our institution

Variable	Low-risk *N* = 634 (65.9)	High-risk *N* = 328 (34.1)	Total *N* = 962	*P*
Sex, N%				
Man	376 (59.3)	189 (57.6)	565 (58.7)	0.629
Woman	258 (40.7)	139 (42.4)	397 (41.3)	
Age, N%				
< 60	353 (55.7)	193 (58.8)	546 (56.8)	0.372
≥ 60	281 (44.3)	135 (41.2)	416 (43.2)	
Laparoscopic surgery, N%				
No	80 (12.6)	62 (18.9)	142 (14.8)	0.012
Yes	554 (87.4)	266 (81.1)	820 (85.2)	
Tumor location, N%				
Right	170 (26.8)	99 (30.2)	269 (28.0)	0.289
Left	464 (73.2)	229 (69.8)	693 (72.0)	
pT stage, N%				
T1-3	634 (100.0)	188 (57.3)	822 (85.4)	< 0.001
T4	0 (0)	140 (42.7)	140 (14.6)	
pN stage, N%				
N1	634 (100.0)	94 (28.7)	728 (75.7)	< 0.001
N2	0 (0)	234 (71.3)	234 (24.3)	
No. LNs harvested, N%				
< 12	65 (10.3)	19 (5.8)	84 (8.7)	0.022
≥ 12	569 (89.7)	309 (94.2)	878 (91.3)	
Histologic grade, N%				
Well/moderately	496 (78.2)	213 (64.9)	709 (73.7)	0.540
Poorly	138 (21.8)	115 (35.1)	253 (26.3)	
Histological type, N (%)				
Adenocarcinoma	540 (85.2)	250 (76.2)	790 (82.1)	0.001
Mucinous or Signet ring cell	94 (14.8)	78 (23.8)	172 (17.9)	
Lymphovascular Invasion, N%				
No	542 (85.5)	214 (65.2)	756 (78.6)	< 0.001
Yes	92 (14.5)	114 (34.8)	206 (21.4)	
Perineural Invasion, N%				
No	505 (79.7)	219 (66.8)	724 (75.3)	< 0.001
Yes	129 (20.3)	109 (33.2)	238 (24.7)	
CDX2, N%				
Positive	619 (97.6)	318 (97.0)	937 (97.4)	0.527
Negative	15 (2.4)	10 (3.0)	25 (2.6)	
MMR status, N%				
dMMR	54 (8.5)	33 (10.1)	87 (9.0)	0.477
pMMR	580 (91.5)	295 (89.9)	875 (91.0)	
Tumor deposits, N%				
No	402 (63.4)	180 (54.9)	582 (60.5)	0.012
Yes	232 (36.6)	148 (45.1)	380 (39.5)	
Timing of adjuvant therapy				
≤ 6 weeks	610 (96.2)	321 (97.9)	931 (96.8)	0.184
> 6 weeks	24 (3.8)	7 (2.1)	31 (3.2)	
Adjuvant chemotherapy, N%				
3 months	373 (58.8)	135 (41.2)	508 (52.8)	< 0.001
3 to 6 months	261 (41.2)	193 (58.8)	454 (47.2)	

*No. LNs* Number of Lymph nodes; *P* < 0.05 is considered statistically significant

### PRFs for OS and DFS in low-risk stage III CC patients

After univariate and multivariable Cox regression analysis for OS and DFS, we found that poorly histological grade (OS: HR = 1.723, 95% CI, 1.005 to 2.951, *P* = 0.048; DFS: HR = 1.634, 95% CI, 1.121 to 2.406, *P* = 0.011), the presence of PNI (OS: HR = 2.273, 95%CI, 1.328 to 3.888, *P* = 0.003; DFS: HR = 2.177, 95% CI, 1.506 to 3.146, *P* < 0.001), and TD (OS: HR = 1.900, 95%CI, 1.151 to 3.136, *P* = 0.012; DFS: HR = 1.683, 95% CI, 1.193 to 2.376, *P* = 0.003) were independent PRFs in low-risk stage III CC patients (Table 2).

**Table 2:** Univariate and multivariate analysis of prognostic factors for OS and DFS in low-risk stage III CC patients from our institution

Variable	OS			
	Univariate analysis		Multivariate analysis	
	HR (95% CI)	*P*	HR (95% CI)	*P*
Sex (Woman vs. Man)	0.461 (0.262–0.812)	0.007	0.527 (0.297–0.933)	0.028
Age (≥ 60 vs. < 60)	2.352 (1.420–3.898)	0.001	2.668 (1.591–4.474)	*P* < 0.001
Laparoscopic surgery (Yes vs. No)	0.499 (0.284–0.879)	0.016	0.528 (0.294–0.948)	0.033
Tumor location (Left vs. Right)	0.706 (0.420–1.188)	0.190	-	-
No. LNs harvested (< 12 vs. ≥ 12)	0.678 (0.345–1.332)	0.259	-	-
Histologic grade (Poorly vs. Well/moderately)	1.583 (0.934–2.681)	0.088	1.723 (1.005–2.951)	0.048
Histological type (Mucinous or Signet ring cell vs. Adenocarcinoma)	1.235 (0.660–2.312)	0.509	-	-
Lymphovascular Invasion (Yes vs. No)	1.537 (0.821–2.879)	0.179	-	-
Perineural invasion (Yes vs. No)	2.169 (1.288–3.652)	0.004	2.273 (1.328–3.888)	0.003
CDX2 (Negative vs. Positive)	2.135 (0.670–6.805)	0.199	-	-
MMR status (pMMR vs. dMMR)	3.300 (0.806–13.507)	0.097	-	0.057
Tumor deposits (Yes vs. No)	1.837 (1.129–2.990)	0.014	1.900 (1.151–3.136)	0.012
Timing of adjuvant therapy (> 6 weeks vs. ≤ 6 weeks)	2.965 (1.281–6.861)	0.011		0.062
Adjuvant chemotherapy (3 to 6 months vs. 3 months)	1.073 (0.658–1.751)	0.777	-	-
Variable	DFS			
	Univariate analysis		Multivariate analysis	
	HR (95% CI)	*P*	HR (95% CI)	*P*
Sex (Woman vs. Man)	0.716 (0.498–1.028)	0.070	-	0.075
Age (≥ 60 vs. < 60)	1.309 (0.930–1.841)	0.122	-	-
Laparoscopic surgery (Yes vs. No)	0.756 (0.474–1.206)	0.241	-	-
Tumor location (Left vs. Right)	0.855 (0.588–1.243)	0.411	-	-
No. LNs harvested (< 12 vs. ≥ 12)	0.770 (0.463–1.282)	0.316	-	-
Histologic grade (Poorly vs. Well/moderately)	1.469 (1.005–2.148)	0.047	1.642 (1.121–2.406)	0.011
Histological type (Mucinous or Signet ring cell vs. Adenocarcinoma)	1.378 (0.892–2.130)	0.149	-	-
Lymphovascular Invasion (Yes vs. No)	1.371 (0.874–2.150)	0.170	-	-
Perineural invasion (Yes vs. No)	2.218 (1.540–3.195)	*P* < 0.001	2.177 (1.506–3.146)	*P* < 0.001
CDX2 (Negative vs. Positive)	1.361 (0.503–3.683)	0.544	-	-
MMR status (pMMR vs. dMMR)	1.533 (0.750–3.133)	0.242	-	-
Tumor deposits (Yes vs. No)	1.731 (1.230–2.435)	0.002	1.683 (1.193–2.376)	0.003
Timing of adjuvant therapy (> 6 weeks vs. ≤ 6 weeks)	1.710 (0.799–3.661)	0.167		
Adjuvant chemotherapy (3 to 6 months vs. 3 months)	1.071 (0.759–1.512)	0.697	-	-

*No. LNs* Number of Lymph nodes; *P* < 0.05 is considered statistically significant

With data from SEER database, 18,547 stage III CC patients were enrolled, including 10,023 (54.0%) with low-risk and 8524 (46.0%) with high-risk (Supplementary Fig. 1). Baseline characteristics of CC patients in the SEER database were listed in Supplementary Table 1. Consistent with results from our institution, poor tumor differentiation, PNI and TD were identified as independent PRFs for OS of CC patients from SEER database (Supplementary Table 2).

### Survival analysis results between low-risk stage III CC patients combined with PRFs and high-risk stage III CC patients

**Fig. 2:**
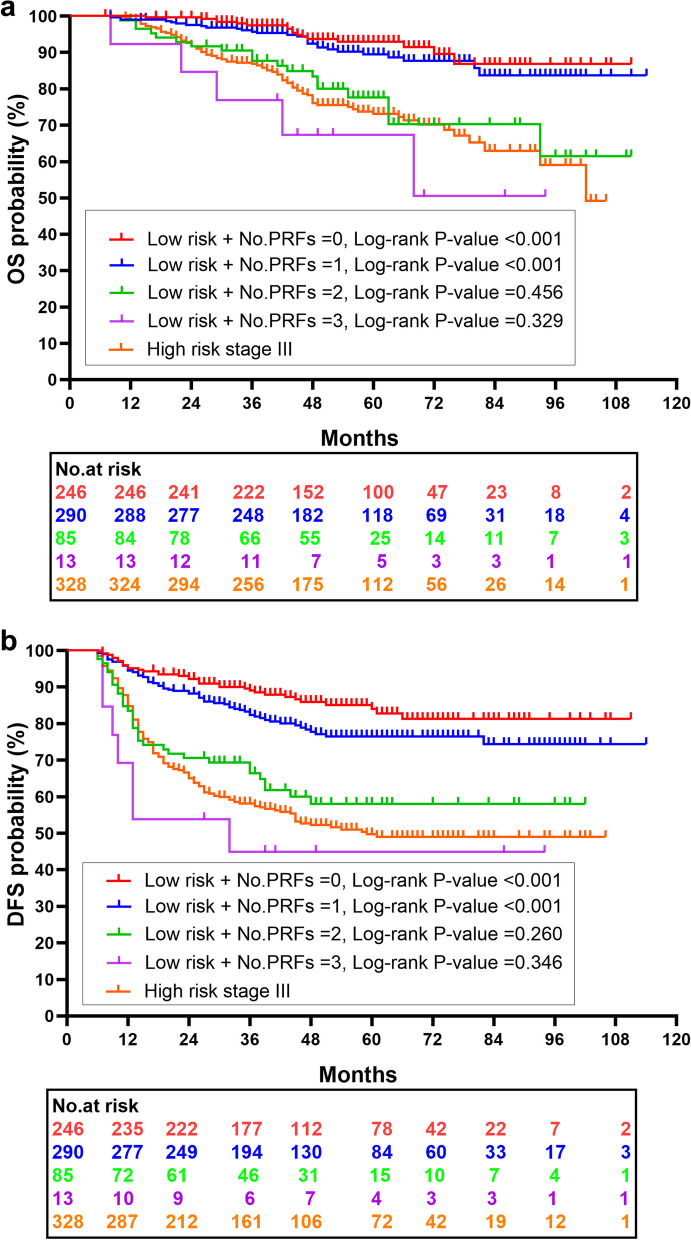
Kaplan–Meier curves comparing OS (**a**) and DFS (**b**) between stage III colon cancer patients with low-risk and high-risk (No., number; PRFs, pathological risk factors. All calculated *p* values are pairwise comparisons with high-risk groups as controls. *P* < 0.05 is considered as statistically significant)

PRFs were used to stratify the low-risk stage III CC patients. In this retrospective cohort study, 246 (38.8%) patients had none of the PRFs, while 290 (45.7%) had 1 PRFs, 85 (13.4%) had 2 PRFs, and 13 (2.1%) had 3 PRFs. The Kaplan–Meier survival curves for OS and DFS stratified by PRFs were shown in Fig. 2. No significant survival difference was observed between low-risk and high-risk stage III CC patients, when their PRFs number was ≥ 2. Then, stratified OS and DFS were calculated using multivariate Cox regression analysis with correction for age and sex. As shown in Table 3, there were significant difference observed between low-risk stage III CC patients with no PRFs and low-risk stage III CC patients with 2 PRFs (OS: HR = 3.871, 95%CI, 2.004–7.479, *P* < 0.001; DFS: HR = 3.479, 95%CI, 2.158–5.610, *P* < 0.001), as well as 3 PRFs (OS: HR = 5.915, 95%CI, 2.623–13.335, *P* < 0.001; DFS: HR = 5.915, 95%CI, 2.623–13.335, *P* < 0.001), and high-risk stage III CC patients (OS: HR = 3.927, 95%CI, 2.317–6.656, *P* < 0.001; DFS: HR = 4.132, 95%CI, 2.858–5.974, *P* < 0.001). These data indicated that prognosis of 15.5% (98/634) low-risk stage III CC patients was similar to that of high-risk stage III CC patients. Therefore, choosing postoperative adjuvant chemotherapy for low-risk stage III CC patients only based on T, N staging system may be inadequate.

**Table 3:** Multivariate analysis of prognostic factors for OS and DFS in low-risk stage III CC patients from our institution

Variable	Multivariate analysis			
	OS		DFS	
	HR (95% CI)	*P*	HR (95% CI)	*P*
Age (≥ 60 vs. < 60)	2.060 (1.469–2.888)	***p*** **< 0.001**	1.360 (1.076–1.719)	**0.010**
Sex (Woman vs. Man)	0.810 (0.573–1.144)	**0.231**	0.868 (0.682–1.104)	**0.249**
Risk group (T1-3N1 + one PRFs vs. T1-3N1 + no PRFs)	1.287 (0.694–2.386)	**0.423**	1.439 (0.945–2.193)	**0.090**
Risk group (T1-3N1 + two PRFs vs. T1-3N1 + no PRFs)	3.871 (2.004–7.479)	***p*** **< 0.001**	3.479 (2.158–5.610)	***p*** **< 0.001**
Risk group (T1-3N1 + three PRFs vs. T1-3N1 + no PRFs)	5.833 (1.953–17.420)	**0.002**	5.915 (2.623–13.335)	***p*** **< 0.001**
Risk group (T4 and/or N2 vs. T1-3N1 + no PRFs)	3.927 (2.317–6.656)	***p*** **< 0.001**	4.132 (2.858–5.974)	***p*** **< 0.001**

*PRFs* Pathological risk factors; *P* < 0.05 is considered statistically significant

After statistical analysis of data from SEER database, low-risk stage III CC patients were stratified into four groups based on number of PRFs: patients with no PRFs (6247, 62.3%), with 1 PRF (2998, 29.9%), with 2 PRFs (700, 6.9%) and those with 3 PRFs (78, 0.7%). The Kaplan–Meier survival curve analysis demonstrated that the prognosis of low-risk stage III CC patients with 3 PRFs was close to that of high-risk stage III CC patients, and patients with less PRFs had a better prognosis than those with more PRFs (Supplementary Fig. 2). Multivariate Cox regression analysis results were illustrated in Supplementary Table 3, there was no significant difference between low-risk stage III CC patients with 3 PRFs (HR = 2.669, 95%CI, 1.898–3.752, *P* < 0.001) and high-risk stage III CC patients (HR = 2.669, 95%CI, 2.503–2.846, *P* < 0.001).

Moreover, we also stratified high-risk stage III CC patients based on the number of PRFs. Similar to the results obtained from low-risk stage III CC patients, the stratified survival analysis results revealed that high-risk stage III CC patients with less PRFs had a better prognosis than those with more PRFs. There was no significant difference for OS between low-risk stage III CC patients with two PRFs and high-risk stage III CC patients with no PRFs, which was better than that of low-risk stage III CC patients with three PRFs (Supplementary Fig. 3). The multivariate Cox regression analysis showed that the HR of low-risk stage III CC patients with two PRFs (HR = 1.857, 95%CI, 1.613–2.139, *P* < 0.001) was similar to that of high-risk stage III CC patients with no PRFs (HR = 1.876, 95%CI, 1.731–2.033, *P* < 0.001) (Supplementary Table 4).

## Discussion

This single-center retrospective cohort study revealed that the presence of PRFs was associated with poor survival of stage III CC patients. The prognosis between low-risk stage III CC patients with no less than 2 PRFs (17.6%, 111/634) and high-risk stage III CC patients was found without significant difference. To further confirm our findings, we collected data of stage III CC patients who received adjuvant chemotherapy from the SEER database. The results suggested that prognosis of some certain low-risk stage III CC patients was underestimated, which was similar to that of high-risk stage III CC patients. Our findings suggest that the selection (both regimen and period) of postoperative adjuvant chemotherapy for low-risk stage III CC patients only based on T/N staging may not be enough.

The IDEA study proposed that stage III CC patients were divided into low-risk (T1-3N1) and high-risk (T4 and/or N2) groups, which suggested that low-risk patients could receive 3 months of adjuvant treatment to reduce chemotherapy-related toxicity [4]. The results from IDEA study revealed that low-risk patients did not benefit from adjuvant chemotherapy for 6 months, and adjuvant CapeOX for 3 months exhibited non-inferiority in DFS from low-risk but not high-risk stage III CC patients. Stage III CC patients are heterogeneous with varied prognosis, which may not be adequately differentiated by the TNM staging system [3]. Previous studies suggested that the 5-year survival rate of stage II CC patients with high-risk (T3N0) was similar to stage III patients with low-risk (T1-2N1) [3]. Besides the T and N stages, pathological features also affect the prognosis of stage III CC patients. The NCCN guidelines recommend adjuvant chemotherapy for high-risk stage II CC patients with PRFs, which were also important for stage III CC patients. Therefore, we aimed to further evaluate the prognosis of stage III CC patients with combined PRFs and optimize risk stratification for low-risk stage III CC patients.

Pathological feature remains essential for post-surgical prognosis, which guides risk stratification and treatment strategy selection for CC patients. Previous studies proposed that tumor-infiltrating lymphocytes (TILs) density and tumor budding were important prognostic variables for stage III CC patients [11–13]. Moreover, the combination of TILs density and tumor budding provided reliable prognostic stratification for T and N risk groups, which was the strongest predictors of DFS in high-risk stage III CC patients [14]. However, comparing with tumor microenvironment characteristics, routine postoperative pathological characteristics are relatively easier to obtain from clinical facilities. Huh et al. [7] reported that LVI and PNI were correlated with worse OS and DFS in stage III CC patients, and patients with positive LVI and PNI were three times more likely to recur than those with negative LVI/PNI. In the study from Liebig et al. [6], PNI was correlated with poorer survival and differentiation as well as higher stage in CRC patients [15]. TD was associated with worse prognosis, as evidenced by the results from the IDEA France study [8]. Furthermore, a study from systematic review and meta-analysis including stage I to IV CRC patients supported the same conclusion [9]. A number of studies also indicated that the number of TD could improve the prognostic prediction accuracy in CRC patients [8, 16, 17]. In the 8th AJCC/TNM staging system [18], although TD was correlated with a poorer prognosis, the number of TD was not included in the TNM staging system. And so far, the origin of TD is unclear. Some studies suggest that there may be three different sources of TD: nerves, blood vessels, and lymph nodes [19–22]. Ignoring the origin of TD and directly adding the number of TD to number of positive lymph nodes may require further investigations. Herein, we considered TD as a qualitative value (i.e., only the presence or absence of TD was considered).

Low-risk stage III CC patients with PRFs may not have a better prognosis than high-risk stage III CC patients. In this study, we identified poor differentiation, PNI and TD as independent unfavorable prognostic factors for DFS and OS in low-risk stage III CC patients. By stratifying by the number of PRFs, low-risk stage III CC patients with two or more PRFs had similar prognosis comparing with high-risk stage III CC patients. We therefore concluded that staging only based on T/N alone is inadequate to guide the therapy selection in stage III CC patients. Due to the relatively small sample size from our center, we retrieved data from the SEER database to further verify our findings.

The analysis results from SEER database revealed that, the prognosis of high-risk stage III CC patients was comparable to that of low-risk stage III CC patients with 3 PRFs. Then two questions were posed: whether there was also difference in prognosis between subgroups among high-risk stage III CC patients and whether the prognosis of low-risk stage III CC patients with 2 PRFs was similar to that of high-risk stage III CC patients with no PRFs. We stratified low-risk and high-risk stage III CC patients separately based on the number of PRFs, and the OS was close between low-risk stage III CC patients with two PRFs and high-risk stage III CC patients with no PRFs. The multivariate Cox regression analysis showed that the HR in low-risk stage III CC patients with 2 PRFs (HR = 1.857, 95%CI, 1.613–2.139, *P* < 0.001) was similar to the HR of high-risk stage III CC patients with no PRFs (HR = 1.876, 95%CI, 1.731–2.033, *P* < 0.001). Based on the analysis results from the SEER database, we consider that at least 7.6% (778/10,023) low-risk stage III CC patients have a similar prognosis comparing with high-risk stage III CC patients. Analysis results from the SEER database further confirmed that selection of postoperative adjuvant chemotherapy for low-risk stage III CC patients only based on T/N stage might not be adequate.

The present study has some limitations. The first is that this study was conducted as a retrospective single-center study and the second is that data from the SEER database lacks some information for DFS analysis. It is necessary to perform a large-scale prospective study to further validate the current conclusion. Since the follow-up period of this study is not long enough, the long-term results may be limited. However, the validation analysis from the SEER database could further supports our finding, and the long-term results need to be updated in future studies.

## Conclusion

In summary, it is not sufficient to guide the selection of postoperative adjuvant chemotherapy for low-risk stage III CC patients only based on T and N stages, and combining PRFs for stratification is necessary. Further large-scale, multicenter studies are needed to determine the PRFs as a reliable factor for prognostic stratification.

### Supplementary Information

**Additional file 1: Supplementary Fig. 1.** Flow chart of patient recruitment from the SEER database in this study.

**Additional file 2: Supplementary Fig. 2.** Kaplan–Meier curves comparing OS between stage III CC patients with low-risk and high-risk from the SEER database (No., number; PRFs, pathological risk factors. All calculated *p*-values are pairwise comparisons with high-risk groups as controls. *P* < 0.05 is considered statistically significant).

**Additional file 3: Supplementary Fig. 3.** Kaplan–Meier curves comparing OS between strata of stage III CC patients from the SEER database after addition of PRFs numbers (No., number; PRFs, pathological risk factors. All calculated *p*-values were pairwise comparisons with high-risk groups without PRFs as controls. *P* < 0.05 is considered statistically significant).

**Additional file 4: Supplementary Table 1.** Characteristics of stage III CC patients with low risk (pT1-3N1) and high risk (pT4 and/or pN2) from the SEER database.

**Additional file 5: Supplementary Table 2.** Univariate and multivariate analysis of prognostic factors for OS in stage III CC patients from the SEER database.

**Additional file 6: Supplementary Table 3.** Multivariate analysis of prognostic factors for OS and DFS in low-risk stage III CC patients from the SEER database.

**Additional file 7: Supplementary Table 4.** Multivariate analysis of prognostic factors for OS and DFS in low-risk stage III CC patients from the SEER database.

## Data Availability

The data generated during the current study are available from the corresponding author on reasonable request.
