# Supplementary figures and images for: Combining pathological risk factors and T, N staging to optimize the assessment for risk stratification and prognostication in low-risk stage III colon cancer
Source: World J Surg Oncol. 2024 Jan 4;22:10. doi: 10.1186/s12957-023-03299-w (PMC10765648; doi:10.1186/s12957-023-03299-w)

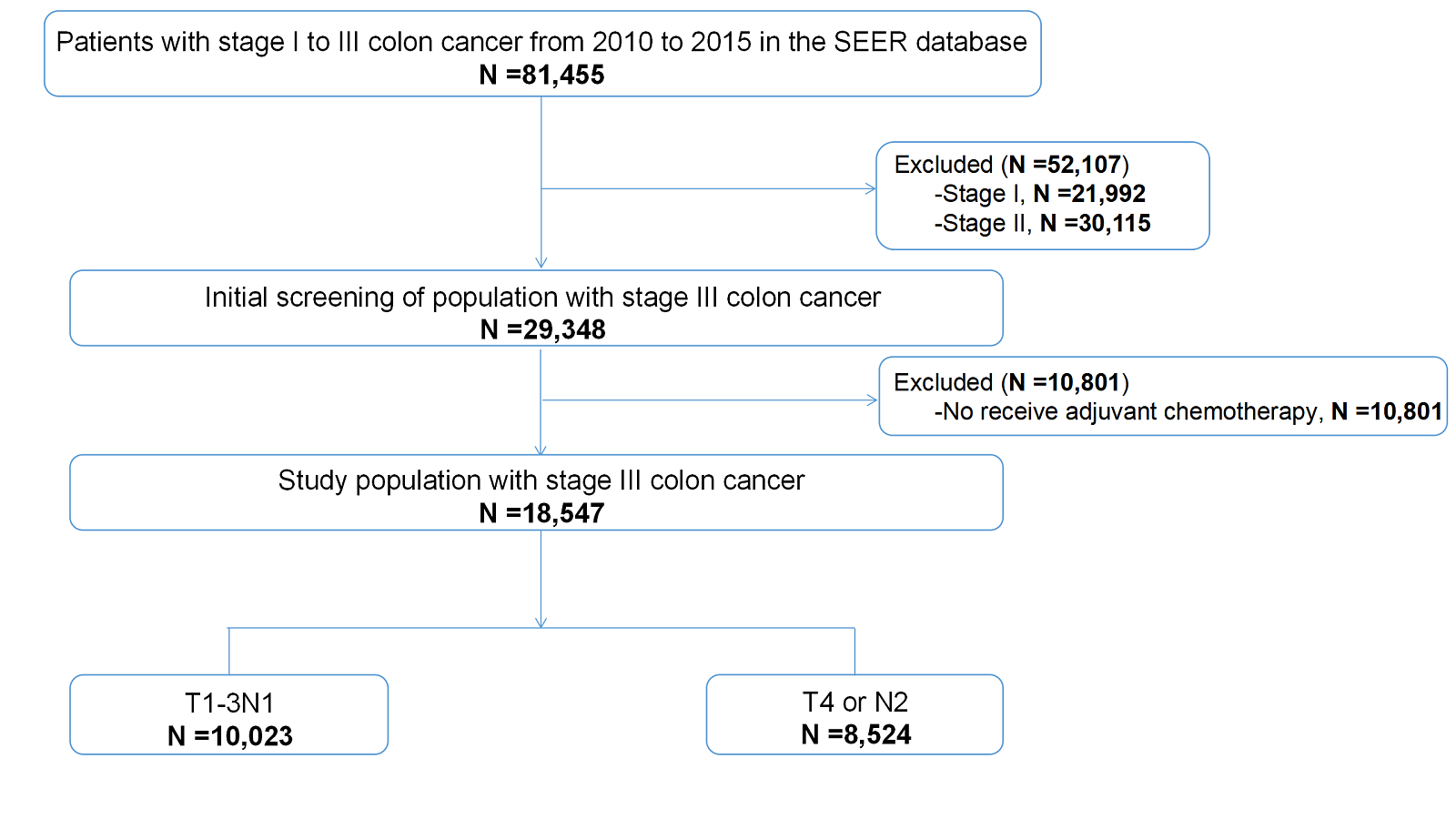

Supplement: Supplementary file 1 — Additional file 1: Supplementary Fig. 1. Flow chart of patient recruitment from the SEER database in this study. [file 12957_2023_3299_MOESM1_ESM.tif]

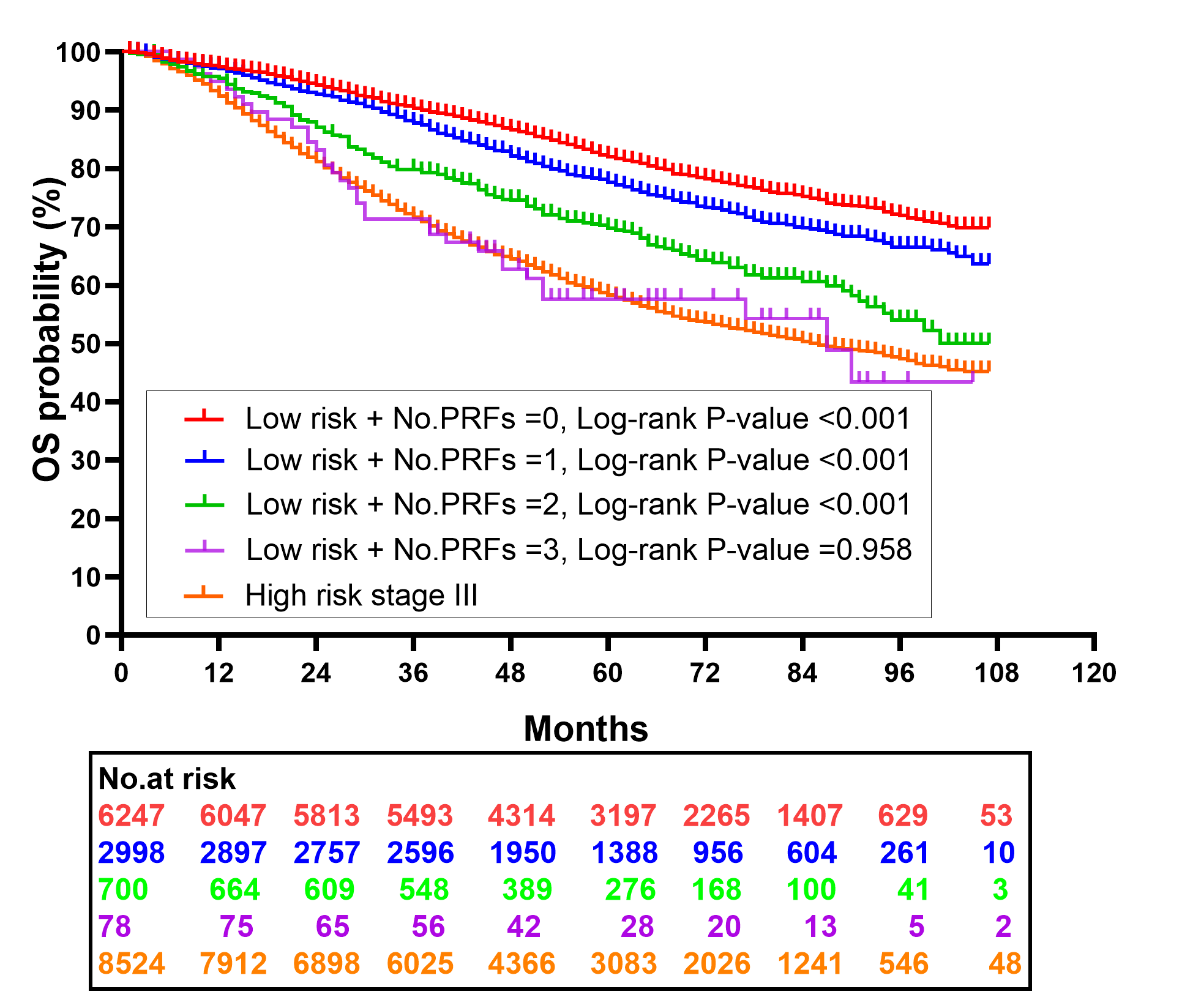

Supplement: Supplementary file 2 — Additional file 2: Supplementary Fig. 2. Kaplan–Meier curves comparing OS between stage III CC patients with low-risk and high-risk from the SEER database (No., number; PRFs, pathological risk factors. All calculated p-values are pairwise comparisons with high-risk groups as controls. P < 0.05 is considered statistically significant). [file 12957_2023_3299_MOESM2_ESM.tif]

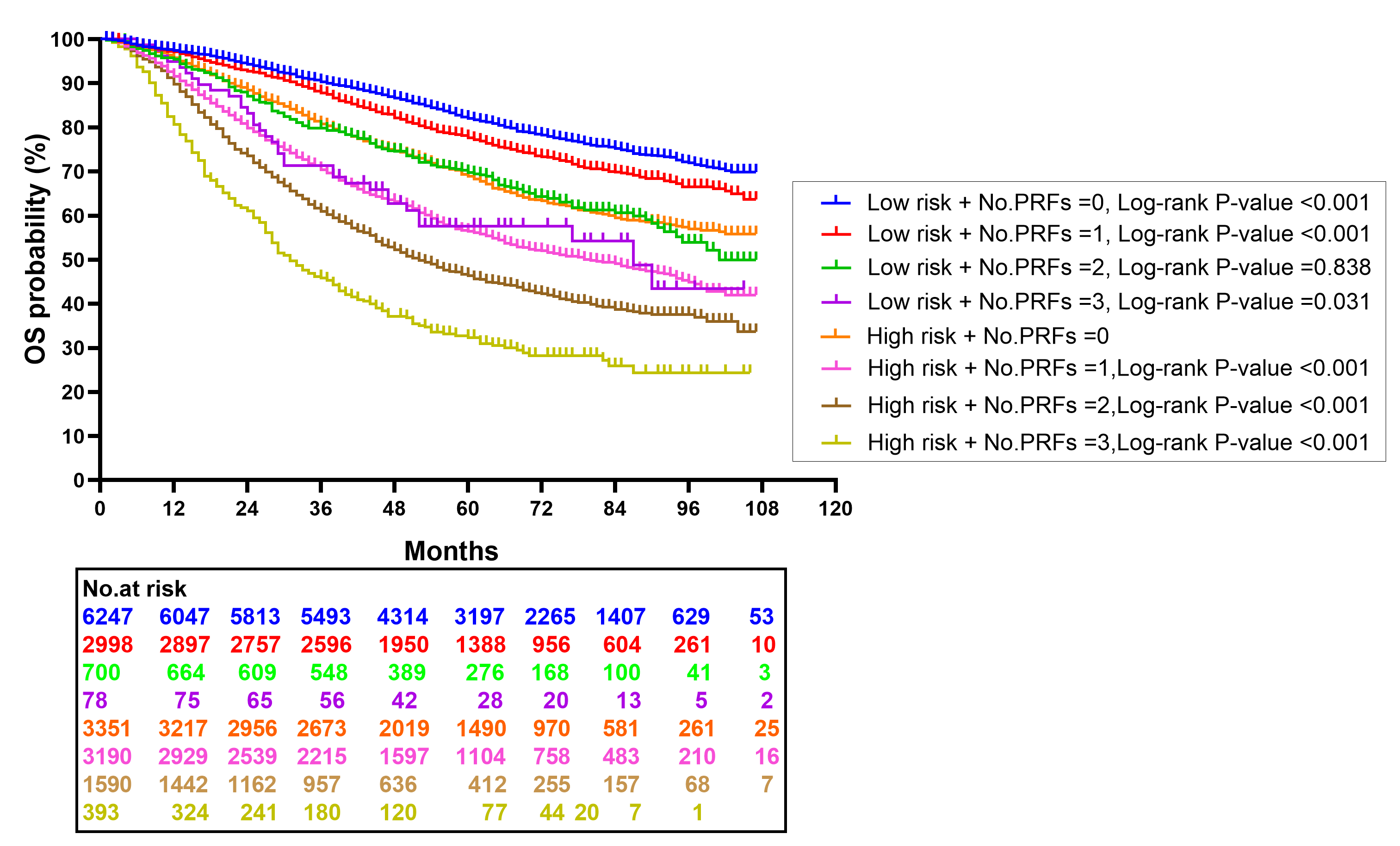

Supplement: Supplementary file 3 — Additional file 3: Supplementary Fig. 3. Kaplan–Meier curves comparing OS between strata of stage III CC patients from the SEER database after addition of PRFs numbers (No., number; PRFs, pathological risk factors. All calculated p-values were pairwise comparisons with high-risk groups without PRFs as controls. P < 0.05 is considered statistically significant). [file 12957_2023_3299_MOESM3_ESM.tif]
