# Supplementary material for: Combining pathological risk factors and T, N staging to optimize the assessment for risk stratification and prognostication in low-risk stage III colon cancer
Source: World J Surg Oncol. 2024 Jan 4;22:10. doi: 10.1186/s12957-023-03299-w (PMC10765648; doi:10.1186/s12957-023-03299-w)
Supplement: Supplementary file 4 — Additional file 4: Supplementary Table 1. Characteristics of stage III CC patients with low risk (pT1-3N1) and high risk (pT4 and/or pN2) from the SEER database. [file 12957_2023_3299_MOESM4_ESM.doc]

**Supplementary Table 1** Characteristics of stage III CC patients with low risk (pT1-3N1) and high risk (pT4 and/or pN2) from the SEER database

| **Variable** | **Low risk**  **N=10,023 (54.0)** | **High risk**  **N=8,524 (46.0)** | **Total**  **N=18,547** | **P** |
| --- | --- | --- | --- | --- |
| Sex, N% |  |  |  |  |
| Female | 4,970 (49.6) | 4,229 (49.6) | 9,199 (49.6) | 0.976 |
| Male | 5,053 (50.4) | 4,295 (50.4) | 9,348 (50.4) |  |
| Age, N% |  |  |  |  |
| <60 | 3,829 (38.2) | 3,427 (40.2) | 7,256 (39.1) | **0.005** |
| ≥60 | 6,194 (61.8) | 5,097 (59.8) | 11,291 (60.9) |  |
| Tumor location, N% |  |  |  |  |
| Left | 4,733 (47.2) | 3,658 (42.9) | 8,391 (45.2) | **P <0.001** |
| Right | 5,290 (52.8) | 4,866 (57.1) | 10,156 (54.8) |  |
| pT stage, N% |  |  |  |  |
| T1-3 | 10,023 (100.0) | 4,624 (54.2) | 14,647 (79.0) | **P <0.001** |
| T4 | 0 (0) | 3,900 (45.8) | 3,900 (21.0) |  |
| pN stage, N% |  |  |  |  |
| N1 | 10,023 (100.0) | 2,080 (24.4) | 12,103 (65.3) | **P <0.001** |
| N2 | 0 (0) | 6,444 (75.6) | 6,444 (34.7) |  |
| No. LNs harvested, N% |  |  |  |  |
| <12 | 1,047 (10.4) | 583 (6.8) | 1,630 (8.8) | **P <0.001** |
| ≥12 | 8,976 (53.1) | 7,941 (93.2) | 16,917 (91.2) |  |
| Histologic grade, N% |  |  |  |  |
| Moderately differentiated; Grade II+ Well differentiated | 8,170 (81.5) | 5,609 (65.8) | 13,779 (74.3) | **P <0.001** |
| Poorly differentiated; Grade III+ Undifferentiated; anaplastic; Grade IV | 1,853 (18.5) | 2,915 (34.2) | 4,768 (25.7) |  |
| Perineural Invasion, N% |  |  |  |  |
| No | 8,879 (88.6) | 6,367 (74.7) | 15,246 (82.2) | **P <0.001** |
| Yes | 1,144 (11.4) | 2,157 (25.3) | 3,301 (17.8) |  |
| Tumor deposits, N% |  |  |  |  |
| No | 8,388 (83.7) | 6,047 (70.9) | 14,435 (77.8) | **P <0.001** |
| Yes | 1,635 (16.3) | 2,477 (29.1) | 4,112 (22.2) |  |

*No. LNs: Number of Lymph nodes; P <0.05 is considered statistically significant*
