# Supplementary material for: Combining pathological risk factors and T, N staging to optimize the assessment for risk stratification and prognostication in low-risk stage III colon cancer
Source: World J Surg Oncol. 2024 Jan 4;22:10. doi: 10.1186/s12957-023-03299-w (PMC10765648; doi:10.1186/s12957-023-03299-w)
Supplement: Supplementary file 5 — Additional file 5: Supplementary Table 2. Univariate and multivariate analysis of prognostic factors for OS in stage III CC patients from the SEER database. [file 12957_2023_3299_MOESM5_ESM.doc]

**Supplementary Table 2**  Univariate and multivariate analysis of prognostic factors for OS in stage III CC patients from the SEER database

| Variable | OS | | | |
| --- | --- | --- | --- | --- |
|  | Univariate analysis | | Multivariate analysis | |
|  | HR  (95% CI) | P | HR  (95% CI) | P |
| Sex  (Male vs. Female) | 1.267 (1.166-1.377) | **P <0.001** | 1.281 (1.178-1.393) | **P <0.001** |
| Age  (≥60 vs. <60) | 1.986 (1.806-2.185) | **P <0.001** | 1.865 (1.693-2.054) | **P <0.001** |
| Tumor location  (Right vs. Left) | 1.388 (1.276-1.511) | **P <0.001** | 1.328 (1.217-1.449) | **P <0.001** |
| No. LNs harvested  (≥12 vs. x＜12) | 0.672 (0.598-0.756) | **P <0.001** | 0.667 (0.592-0.750) | **P <0.001** |
| Histologic grade  (Poorly ^a^ vs. Well/moderately ^b^) | 1.292 (1.169-1.428) | **P <0.001** | 1.211 (1.095-1.339) | **P <0.001** |
| Perineural invasion  (Yes vs. No) | 1.390 (1.235-1.563) | **P <0.001** | 1.327 (1.178-1.495) | **P <0.001** |
| Tumor deposits  (Yes vs. No) | 1.540 (1.391-1.704) | **P <0.001** | 1.495 (1.349-1.657) | **P <0.001** |

*a:* Poorly differentiated; Grade III+ Undifferentiated; anaplastic; Grade IV

b: Moderately differentiated; Grade II+ Well differentiated

*No. LNs: Number of Lymph nodes*

*P <0.05 is considered statistically significant*
