# Supplementary material for: Combining pathological risk factors and T, N staging to optimize the assessment for risk stratification and prognostication in low-risk stage III colon cancer
Source: World J Surg Oncol. 2024 Jan 4;22:10. doi: 10.1186/s12957-023-03299-w (PMC10765648; doi:10.1186/s12957-023-03299-w)
Supplement: Supplementary file 6 — Additional file 6: Supplementary Table 3. Multivariate analysis of prognostic factors for OS and DFS in low-risk stage III CC patients from the SEER database. [file 12957_2023_3299_MOESM6_ESM.doc]

**Supplementary Table 3**  Multivariate analysis of prognostic factors for OS and DFS in low-risk stage III CC patients from the SEER database

| Variable | Multivariate analysis | |
| --- | --- | --- |
|  | OS | |
|  | HR  (95% CI) | P |
| Age (≥60 vs. <60) | 1.726 (1.632-1.825) | **p<0.001** |
| Sex (Woman vs. Man) | 1.108 (1.053-1.166) | **p<0.001** |
| Risk group (T1-3N1 + one PRFs vs. T1-3N1 + no PRFs) | 1.251 (1.142-1.370) | **p<0.001** |
| Risk group (T1-3N1 + two PRFs vs. T1-3N1 + no PRFs) | 1.854 (1.610-2.135) | **p<0.001** |
| Risk group (T1-3N1 + three PRFs vs. T1-3N1 + no PRFs) | 2.669 (1.898-3.752) | **p<0.001** |
| Risk group (T4 and/or N2 vs. T1-3N1 + no PRFs) | 2.669 (2.503-2.846) | **p<0.001** |

*PRFs: Pathological risk factors; P <0.05 is considered statistically significant.*
