# Supplementary material for: Combining pathological risk factors and T, N staging to optimize the assessment for risk stratification and prognostication in low-risk stage III colon cancer
Source: World J Surg Oncol. 2024 Jan 4;22:10. doi: 10.1186/s12957-023-03299-w (PMC10765648; doi:10.1186/s12957-023-03299-w)
Supplement: Supplementary file 7 — Additional file 7: Supplementary Table 4. Multivariate analysis of prognostic factors for OS and DFS in low-risk stage III CC patients from the SEER database. [file 12957_2023_3299_MOESM7_ESM.doc]

**Supplementary Table 4**  Multivariate analysis of prognostic factors for OS and DFS in low-risk stage III CC patients from the SEER database

| Variable | Multivariate analysis | |
| --- | --- | --- |
|  | OS | |
|  | HR  (95% CI) | P |
| Age (≥60 vs. <60) | 1.734 (1.640-1.834) | **p<0.001** |
| Sex (Woman vs. Man) | 1.120 (1.064-1.178) | **p<0.001** |
| Risk group (T1-3N1 + one PRFs vs. T1-3N1 + no PRFs) | 1.252 (1.143-1.371) | **p<0.001** |
| Risk group (T1-3N1 + two PRFs vs. T1-3N1 + no PRFs) | 1.857 (1.613-2.139) | **p<0.001** |
| Risk group (T1-3N1 + three PRFs vs. T1-3N1 + no PRFs) | 2.675 (1.903-3.761) | **p<0.001** |
| Risk group (T4 and/or N2 + no PRFs vs. T1-3N1 + no PRFs) | 1.876 (1.731-2.033) | **p<0.001** |
| Risk group (T4 and/or N2 + one PRFs vs. T1-3N1 + no PRFs) | 2.811 (2.606-3.033) | **p<0.001** |
| Risk group (T4 and/or N2 + two PRFs vs. T1-3N1 + no PRFs) | 3.856 (3.534-4.209) | **p<0.001** |
| Risk group (T4 and/or N2 + three PRFs vs. T1-3N1 + no PRFs) | 6.217 (5.444-7.100) | **p<0.001** |

*PRFs: Pathological risk factors; P <0.05 is considered statistically significant.*
